# Improving the measurement properties of the Amyotrophic Lateral Sclerosis Functional Rating Scale-Revised (ALSFRS-R): deriving a valid measurement total for the calculation of change

**DOI:** 10.1080/21678421.2024.2322539

**Published:** 2024-03-01

**Authors:** Carolyn A Young, Amina Chaouch, Christopher J Mcdermott, Ammar Al-Chalabi, Suresh K Chhetri, Kevin Talbot, Andrea Malaspina, Roger Mills, Alan Tennant

**Affiliations:** 1 Walton Centre NHS Foundation Trust, Liverpool, UK; 2 Department of Pharmacology and Therapeutics, University of Liverpool, UK; 3 Greater Manchester Centre for Clinical Neurosciences, Salford, UK; 4 Sheffield Institute for Translational Neuroscience, Sheffield, UK; 5 Maurice Wohl Clinical Neuroscience Institute, Department of Basic and Clinical Neuroscience, King’s College London, London, UK; 6 Department of Neurology, King’s College Hospital, London, UK; 7 Lancashire Teaching Hospital, Preston, UK; 8 Nuffield Department of Clinical Neurosciences, University of Oxford, Oxford, UK; 9 UCL Queen Square Institute of Neurology, London, UK, and; 10 Leeds Institute of Rheumatic and Musculoskeletal Medicine, University of Leeds, UK

**Keywords:** Amyotrophic Lateral Sclerosis Functional Rating Scale-Revised, Trajectories of Outcome in Neurological Conditions-ALS, Rasch, outcome measurement, disability

## Abstract

**Background:**

The Amyotrophic Lateral Sclerosis Functional Rating Scale-Revised (ALSFRS-R) total score is a widely used measure of functional status in Amyotrophic Lateral Sclerosis/Motor Neuron Disease (ALS), but recent evidence has raised doubts about its validity. The objective was to examine the measurement properties of the ALSFRS-R, aiming to produce valid measurement from all 12 scale items.

**Method:**

Longitudinal ALSFRS-R data were collected between 2013-2020 from 1120 people with ALS recruited from 35 centers, together with other scales in the Trajectories of Outcomes in Neurological Conditions-ALS (TONiC-ALS) study. The ALSFRS-R was analyzed by confirmatory factor analysis (CFA), Rasch Analysis (RA) and Mokken scaling.

**Results:**

No definite factor structure of the ALSFRS-R was confirmed by CFA. RA revealed the raw score total to be invalid even at the ordinal level because of multidimensionality; valid interval level subscale measures could be found for the Bulbar, Fine-Motor and Gross-Motor domains but the Respiratory domain was only valid at an ordinal level. All four domains resolved into a single valid, interval level measure by using a bifactor RA. The smallest detectable difference was 10.4% of the range of the interval scale.

**Conclusion:**

A total ALSFRS-R ordinal raw score can lead to inferential bias in clinical trial results due to its non-linear nature. On the interval level transformation, more than 5 points difference is required before a statistically significant detectable difference can be observed. Transformation to interval level data should be mandatory in clinical trials.

## Introduction

Amyotrophic lateral sclerosis (ALS), also known as Motor Neuron Disease (MND), is an incurable, neurodegenerative condition where inexorable progression leads to severe disability. In a meta-analysis of 115 studies involving 55,169 ALS patients over 24 years, change in functional status was found to predict survival (1). It follows that the measurement of change in functioning is crucial not only to follow the progression of the disease, but also to inform clinical management. Furthermore, many trials employ change in functioning as a study endpoint.

In a review of 125 clinical trials, the revised Amyotrophic Lateral Sclerosis Functional Rating Scale (ALSFRS-R) was used in 47 of 51 studies employing a functional rating scale as the primary outcome measure (2). The ALSFRS-R was designed to overcome the weakness of the original which granted disproportionate weighting to limb and bulbar, as compared to respiratory, dysfunction; to correct this the revision added items for dyspnea, orthopnoea, and the need for ventilatory support (3). The ALSFRS-R consists of 12 items each with five levels of severity, the most disabled level is assigned a score of 0 and the least 4, so the total score ranges from 0-48 with a higher score representing better functioning.

Despite its widespread use, concern has been raised about the structural validity of the ALSFRS-R and in particular the validity of the total score. One study using the Rasch model found that the ALSFRS-R failed to satisfy rigorous measurement standards and should be considered as a profile of mean scores from three different domains (Bulbar, Motor and Respiratory functions) rather than a total score (4). Another study found that the interpretation of a total raw score of ALSFRS-R was hampered by ambiguities due to the different metric properties of the three domains aggregated in the scale (5). Two studies found that confirmatory-factor analysis (CFA) supported a four-factor structure (Bulbar, Gross-Motor, Fine-Motor, and Respiratory domains) rather than a total score (6, 7). Recently it has been argued that ignoring the multidimensional structure of the ALSFRS-R total score could have negative consequences for ALS clinical trials and that treatment benefit should be analyzed at the subscale level (8). In addition, the use of change scores to assess the efficacy of a treatment to alter functioning, whether from a total or subscale score, can only be validly computed for interval level data (9–12), hence the importance of being able to generate interval level measurement from the ALSFRS-R.

Consequently, this study seeks to examine the measurement structure of the ALSFRS-R in a large population within the framework of both classical (factor analytic) and Rasch Measurement Theory, to verify its measurement properties and determine if an interval scale transformation is viable (13, 14).

## Methods

### Main sample and data collection

Participants with ALS were recruited into the Trajectories of Outcomes in Neurological Conditions-ALS (TONiC-ALS) study from 35 specialist clinics across the United Kingdom between 2013 and 2020 (15). Patient reported outcome measures were collected for depression and anxiety using Modified-Hospital Anxiety and Depression scale (M-HADS) (16); health status using EQ-5D-5L (17); as well as a lay language self-administered ALSFRS-R based on earlier validation work (18). The TONiC-ALS version corresponds with the original Cedarbaum wording and European Network to Cure ALS (ENCALS) and Northeast Amyotrophic Lateral Sclerosis Consortium (NEALS) versions apart from use of lay language and some minor changes, described in the Supplementary File. Severity was graded using the King’s ALS staging system (19). All participants were eligible for follow-up with repeat packs at least 4 months apart. Ethical approval was granted from research committees (reference 11/NW/0743).

### Calibration, training and validation samples

A calibration sample of 1000 participants was randomized into ‘training’ and ‘validation’ samples of 500 participants for use in the CFA and Rasch analysis (details in Supplementary File).

### Confirmatory factor analysis (CFA)

A CFA was applied to both the three- and four- factor solutions. The four-factor solution comprises Bulbar, Gross-Motor, Fine-Motor, and Respiratory domains whereas the three-factor version combines both Motor into a single Limb domain (details in Supplementary File).

### Rasch analysis

Data from the ALSFRS-R were also fit to the Rasch measurement model, to evaluate the scale’s construct validity and to test if it was possible to provide interval level latent estimates for parametric analysis (13, 14). Due to the known multidimensional nature of the scale, a bi-factor approach was used (20, 21). This approach seeks to identify the variance in the data that is common across subscales (i.e., Bulbar, Limb and Respiratory), discarding that which is unique to each subscale. Each subscale is referred to as a ‘testlet’, comprising the summed score of the subscale. Consequently, three testlets are fitted to the Rasch model. In RUMM2030, the software automatically produces a bi-factor solution (14, 22). An interval scale latent estimate is then derived from this common variance, often thought of as the ‘first common factor’. The solution must satisfy the full requirements of the Rasch model as described in Supplementary File; each testlet is treated as an item and invariance (Differential Item Functioning) of the scale is tested for age, gender, onset type and duration. Levels of acceptable fit to the Rasch model are provided in the table of results. Where acceptable fit was achieved, a transformation of the raw score total to an interval level metric of 0.0–48.0 is performed. Should an interval level solution not be possible through Rasch analysis, then ordinal level validity would be tested by Mokken scaling (13, 14, 23).

**Table 1.** Demographic and clinical characteristics of the different samples.

Sample/Characteristics	Main	Calibration
Mean Age (SD)	65.0 (10.7)	65.7 (10.5)
% Male	60.3	63.1
Months since diagnosis – Median	9	14
Interquartile Range	3–24	7–30
Months since diagnosis – Mean (SD)	23.3 (41.6)	29.8 (45.4)
% Onset Type		
Bulbar	25.9	23.8
Limb	68.3	71.9
Respiratory	2	1.7
Unknown*	3.8	2.6
ALSFRS-R – Median	35	32
Interquartile Range	28–39	25–38
Range	1–48	1–48
% King’s Stage 3–4	54.7	63.8
*N*	1120	1000

*Onset type at diagnosis, of limb, bulbar or respiratory, not specified (there was no option for mixed onset).

### Additional analyses

Reliability was determined from Cronbach’s alpha and the Person Separation Index (PSI) (details in Supplementary File). Using interval level data, the Standard Error of Measurement (SEM) was calculated as SD*√(1-reliability). The Smallest Detectable Difference (SDD), which provides a value for the minimum difference that must be observed to be sure that any observed difference is real, rather than possibly due to measurement error, was calculated as ±1.96*√2*SEM.

## Results

### Samples

The demographic and clinical characteristics of the samples are shown in Table 1.

**Table 2.** Fit of ALSFRS-R (sub)scale to the Rasch model. Each sample N = 500.

Scale	Sample	SD of Fit residuals		Chi-Square			Reliability		Unidimensionality		DIF present
		Items	Persons	Value	df	p	PSI	Alpha	% *t*-test	ECV	
Bulbar	Training	1.223	0.957	29.1	24	0.177	0.69	0.87	1.43	–	No
	Validation	1.064	0.872	32.4	24	0.117	0.71	0.89	3.25	–	O-speech
Fine-Motor	Training	1.669	0.891	30.9	24	0.157	0.80	0.88	2.43	–	G-handwriting
	Validation	1.382	0.868	21.1	24	0.634	0.80	0.88	2.20	–	No
Gross-Motor	Training	3.766	0.950	9.3 ^	4	0.054	0.72	0.71	1.35	0.83	No
	Validation	3.748	0.826	11.6 ^	4	0.020	0.69	0.70	1.14	0.83	G
Limb	Training	0.572	0.871	16.5 ^	16	0.417	0.71	0.80	1.1	0.89	G
	Validation	0.152	0.934	44.0 ^	16	0.002	0.69	0.78	1.6	0.88	G
Respiratory	Training	0.864	0.745	47.3	20	<0.001	0.54	0.82	0.3	–	No
	Validation	1.157	0.659	126.0	18	<0.001	0.65	0.84	1.3	–	D-respiratory support
Total	Training	0.301	0.798	40.9 ^	36	0.279	0.59	0.67	0.9	0.80	O
	Validation	0.013	0.817	32.8 ^	37	0.664	0.64	0.72	1.1	0.85	O
	Full Sample	0.238	0.824	42.3 ^	38	0.291	0.62	0.70	1.3	0.82	No
**Ideal Values**		1	1			>0.01	>0.7	>0.7	<5%	>0.85	

Abbreviations: df: degrees of freedom; PSI: Person Separation Index; Alpha: Cronbach’s Alpha; ECV: Expected Common Variance (only available for testlet analysis); DIF: Differential Item Functioning - G Gender, O Onset, D Duration;  ^ Conditional Test of Fit.

### Confirmatory factor analysis

**Figure 1.**
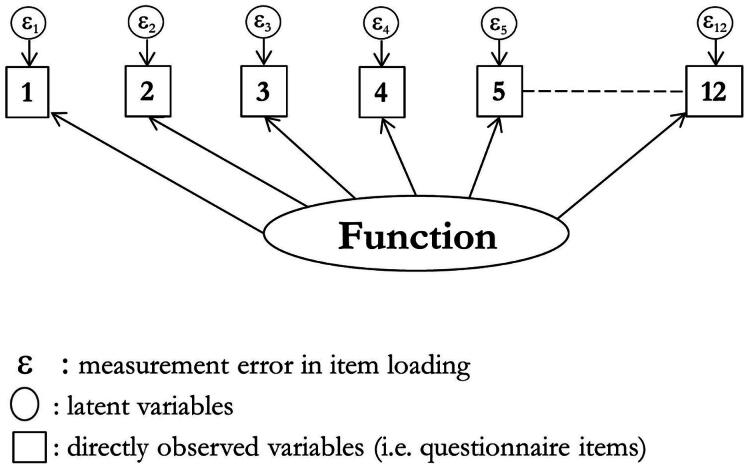
Schematic diagram of 12 item confirmatory factor analysis.

The three-domain version (Figure 1) failed a CFA in the training sample, even when most items had errors correlated (*χ*^2^ 123.8 (df 42): *p* ≤ 0.001; RMSEA 0.062, CFI 0.977, TLI 0.964). This was replicated in the validation sample (χ^2^ 103.4 (df 42): *p* ≤ 0.001; RMSEA 0.054, CFI 0.983, TLI 0.974).

**Figure 2.**
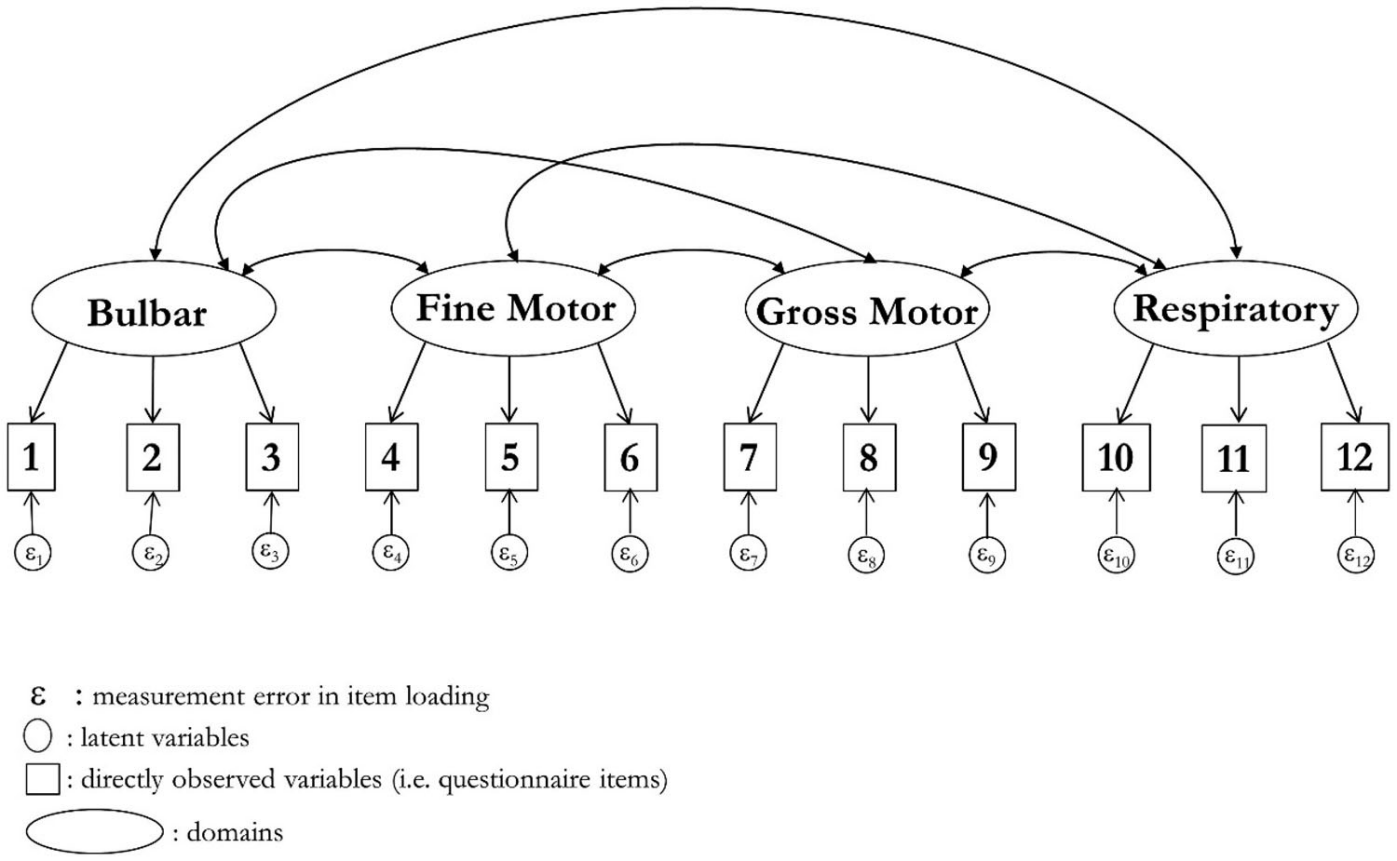
The four domain confirmatory factor analysis.

The four-domain approach also failed a CFA in the training sample (Figure 2) (*χ*^2^ 361.2 (df 48): *p* ≤ 0.001; RMSEA 0.114, CFI 0.914, TLI 0.882). Considerable item local dependency existed and, allowing for correlated errors, model fit was poor although approximate fit statistics were improved (χ^2^ 102.6 (df 42): *p* ≤ 0.001; RMSEA 0.054, CFI 0.983, TLI 0.974). Cross-loading was present suggesting inconsistency of the four-domain structure. The validation sample confirmed this (χ^2^ 116.1 (df 42): *p* ≤ 0.001; RMSEA 0.059, CFI 0.980, TLI 0.968).

### Rasch analysis

**Figure 3.**
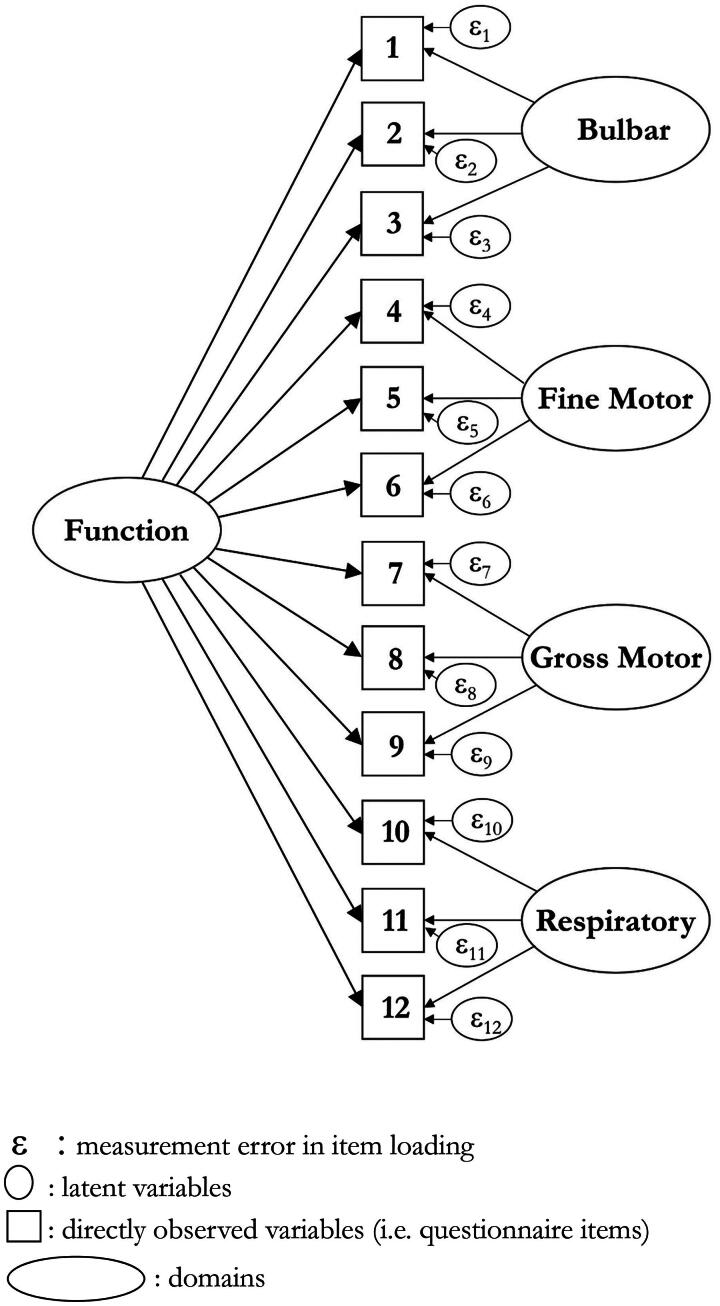
Representation of the bi-factor model as applied within the Rasch measurement framework.

The data from subscales and the total score were fit to the Rasch model (Table 2). A total score achieved satisfactory fit to the model under a bi-factor solution, based upon two testlets, one with the Bulbar and Respiratory subscales, and the other containing Limb (Fine-Motor and Gross-Motor) domains (Figure 3). There was intermittent lack of invariance (i.e. DIF) across different domains for different grouped factors, but for the total score, only onset type remained. However, controlling for DIF (split and unsplit solution) found an effect size of 0.019, so invariance by onset group was considered trivial and disregarded.

**Figure 4.**
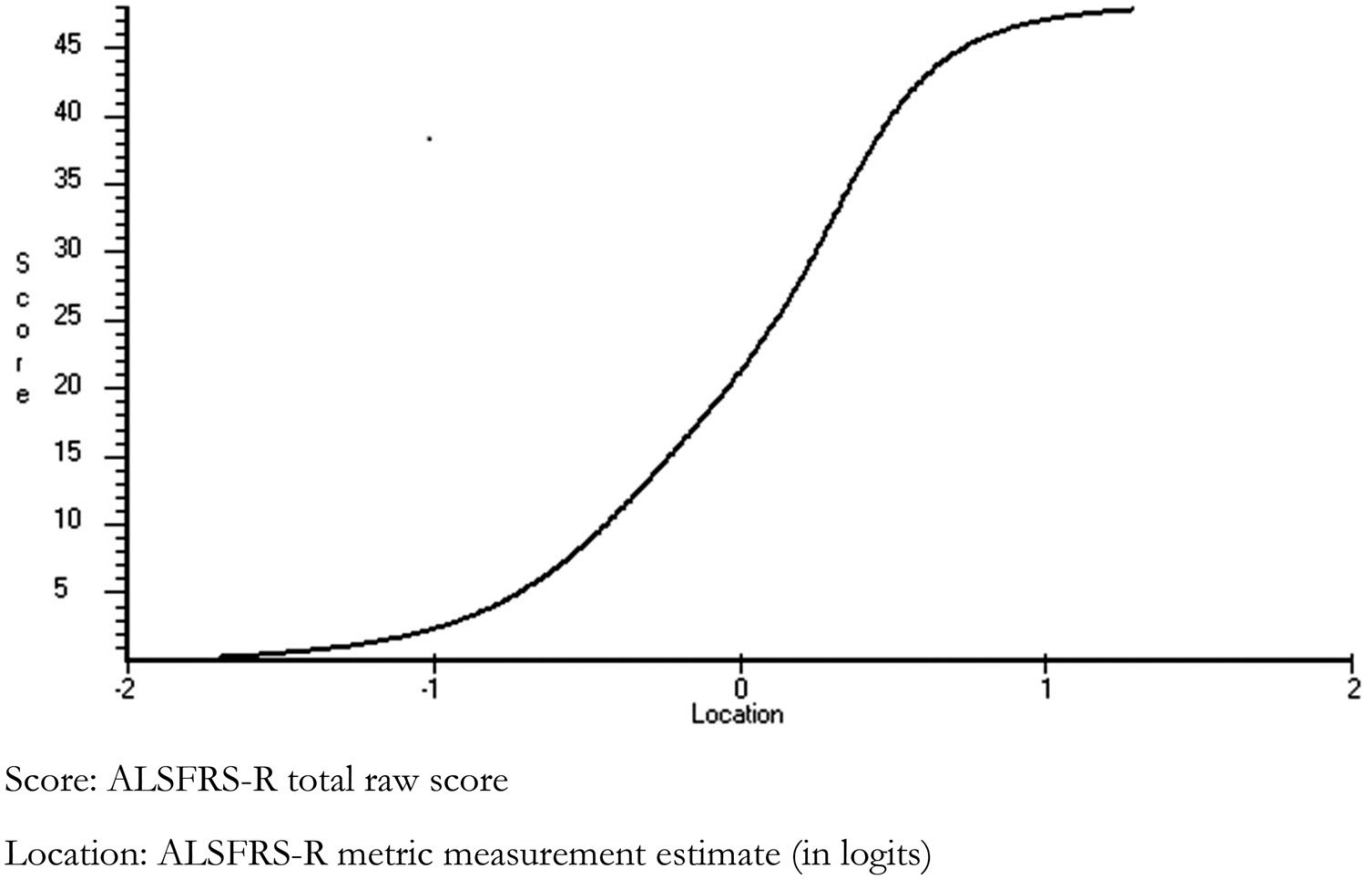
Raw score to metric ogive for ALSFRS-R Total.

The relationship between raw and interval scores is shown in Figure 4. This is based on equating each raw score to its logit estimate where data fit the model, which provides a simple transformation based on the raw score. Providing all 12 items of the ALSFRS-R are completed, the ordinal raw total score can be transformed to the interval level metric suitable for parametric analyses, such as change scores. Interval level equivalents can also be read for Bulbar, Gross-Motor, Fine-Motor or Limb subscales, the last combining Gross- and Fine-Motor. A transformation table of raw scores to interval level metrics, based on this solution and subscale specific solutions using the total sample of 1120 subjects, is given in Table 3. The Respiratory subscale cannot generate interval level measurement, but gave a Loevinger H Coefficient of 0.752 from Mokken scale analysis, suggesting the raw ordinal subscale score is valid.

**Table 3.** Transformation of raw scores to interval scale metric.

Raw score	Total	Bulbar	Fine-motor	Gross-motor	Limb
48	48.0				
47	41.5				
46	37.6				
45	35.2				
44	33.5				
43	32.2				
42	31.1				
41	30.1				
40	29.2				
39	28.4				
38	27.7				
37	27.0				
36	26.3				
35	25.7				
34	25.0				
33	24.4				
32	23.8				
31	23.2				
30	22.7				
29	22.1				
28	21.6				
27	21.1				
26	20.6				
25	20.1				
24	19.6				24.0
23	19.2				21.0
22	18.7				19.1
21	18.3				17.8
20	18.0				16.8
19	17.6				16.0
18	17.2				15.2
17	16.8				14.5
16	16.5				13.8
15	16.1				13.1
14	15.7				12.4
13	15.3				11.7
12	15.0	12.0	12.0	12.0	11.0
11	14.6	10.4	10.4	10.8	10.3
10	14.1	9.1	9.1	9.8	9.7
9	13.7	8.1	8.0	9.1	9.0
8	13.2	7.1	7.1	8.5	8.4
7	12.6	6.1	6.2	7.7	7.8
6	12.0	5.3	5.4	6.9	7.1
5	11.3	4.5	4.6	6.0	6.5
4	10.4	3.8	4.0	5.1	5.8
3	9.3	3.0	3.3	4.1	5.0
2	7.7	2.3	2.5	3.0	3.9
1	4.9	1.3	1.4	1.7	2.4
0	0.0	0.0	0.0	0.0	0.0

High score on ALSFRS-R indicates less disability.

**Instructions for use of the nomogram**.

Providing the respondent has answered all the items, take the raw score and look across to the interval scale estimate for the relevant (sub)scale.

For example, if you are converting the total ALSFRS-R, a raw score of 30 would give a standardized metric of 22.7.

A raw total ALSFRS-R score of 10 would give a standardized metric of 14.1.

A raw bulbar score (speech, salivation, swallowing) of 10 gives a standardized metric of 9.1.

A raw fine-motor score (handwriting, cutting food, dressing) of 10 gives a standardized metric of 9.1.

A raw gross-motor score (turning in bed, walking, stairs) of 10 gives a standardized metric of 9.8.

### Descriptive analysis using interval level metric scores

The age-sex specific baseline estimates in the full sample for the ALSFRS-R interval level scores are given in Table 4, along with duration, health status (EQ-5D-5L), and percent with bulbar onset and King’s Stage greater than 2. The overall mean of the ALSFRS-R metric was 25.0 (SD 5.7), equating to raw score of 34 on the transformation table. Standard Error of Measurement (SEM) was 1.80, Smallest Detectable Difference (SDD) was 5.0, which represents 10.4% of the operational scale width. There was little variation over age-sex specific groups.

**Table 4.** Age-sex specific age, duration, ALSFRS-R interval level (metric) total, and EQ-5D-5L utility value in full sample at baseline (*N* = 1107).

Age	Sex	Age (Mean)	Duration/ Months (Median)	ALSFRS-R Metric (Mean)	EQ-5D-5L (Mean)	% Bulbar Onset	% King’s Stage >2	*N*
Under 50	Male	43.0	10	25.4	0.609	5.8	51.8	56
	Female	42.4	16	24.1	0.491	10.8	48.6	36
	Total	42.8	10	24.9	0.563	7.9	50.5	92
50-54	Male	52.3	9	26.0	0.580	21.2	43.4	53
	Female	52.4	8	25.4	0.608	17.7	47.2	36
	Total	52.3	9	25.8	0.591	19.8	44.9	89
55-59	Male	57.3	11	25.5	0.610	22.5	51.9	81
	Female	57.3	8	26.3	0.562	40.0	41.7	36
	Total	57.3	9	25.8	0.596	27.8	48.7	117
60-64	Male	62.2	8	25.0	0.574	17.5	54.0	125
	Female	62.2	9	24.0	0.565	41.1	59.0	61
	Total	62.2	8	24.7	0.571	25.0	55.6	186
65-69	Male	67.1	9	25.0	0.596	23.1	53.2	135
	Female	67.1	7	24.7	0.617	34.7	58.6	97
	Total	67.1	9	24.9	0.605	28.0	55.5	232
70-74	Male	71.9	10	25.7	0.619	22.4	50.9	112
	Female	71.9	8	24.7	0.623	43.3	60.0	90
	Total	71.9	9	25.2	0.621	32.0	55.0	202
75+	Male	78.9	7	24.8	0.606	25.4	63.0	106
	Female	80.0	6.5	23.7	0.590	43.2	62.7	83
	Total	79.4	7	24.6	0.601	33.5	62.5	189
TOTAL		65.0	9	25.0	0.696	26.7	54.7	1107

Abbreviation: ALSFRS-R: Amyotrophic Lateral Sclerosis Functional Rating Scale-Revised.

The interval level total and subscale measures, by onset type, are shown in Table 5. As expected, those with bulbar onset had the lowest Bulbar subscale (lower scores indicate worse functioning) and higher Limb function, while those with limb onset had higher Bulbar functioning and lower Limb function. All scales showed a significant difference for onset type, for example, limb onset showed a higher score than respiratory onset (ANOVA F 6.98 (df2); *p* ≤ 0.001), although the effect size was just 0.144, considered trivial.

**Table 5.** Total and subscale interval level scores from ALSFRS-R by onset type (*N* = 1107).

Onset/Scale	Total	Subscales			
		Bulbar	Fine-Motor	Gross-Motor	Limb
Bulbar	24.3	4.8	8.4	8.7	15.4
Limb	25.3	9.5	6.7	6.8	12.0
Respiratory	21.5	8.7	6.1	6.8	11.6
Total	25.0	8.2	7.1	7.3	12.9
**Range**	**0–48**	**0–12**	**0–12**	**0–12**	**0–24**

All domains show significant difference across onset groups (ANOVA *p* ≤ 0.001).

High score represents high functioning.

The interval level ALSFRS-R total showed a strong significant gradient across King’s Staging (ANOVA *p* ≤ 0.001). There was a significant increase in disability (downward gradient) across grouped duration since diagnosis (ANOVA F 33.6 (df 3); *p* ≤ 0.001). The effect size of the difference in the ALSFRS-R metric across the shortest duration group (<7 months) and the longest duration group (30+ months) was 0.683, rated medium. This was slightly larger than the corresponding effect size for EQ-5D-5L, at 0.615.

Functioning was found to be associated with the level of depression. Those without any depression on the M-HADS-D at baseline had an interval level ALSFRS-R score of 26.26 (SD 5.60) compared with those with probable depression showing worse functioning at 21.73 (SD 5.39) (ANOVA F 58.87 (df 2); *p* ≤ 0.001). The effect size of this difference was 0.768. A similar, but less strong, effect (0.452) was shown for anxiety.

A multi-level mixed effects regression in the longitudinal data showed that lower ALSFRS-R scores, indicating greater disability, were associated with being female, compared to male, and both bulbar and respiratory onset compared to limb onset (Table 6). In addition, the longer the duration, the lower the ALSFRS-R score.

**Table 6.** Multi-level mixed regression.

ALSFRS-R	Coef.	St.Error		*t*-value	*p*-value	[95% Confidence Interval]		Sig
Age	−.001	.005		−0.11	.911	−.01	.009	
Female	−.718	.128		−5.59	.000	−.97	−.467	***
Married	−.468	.305		−1.53	.125	−1.067	.13	
Onset (limb reference)								
Bulbar	−1.089		.262	−4.15	.000	−1.602	−.575	***
Respiratory	−3.737		.32	−11.69	.000	−4.364	−3.111	***
Duration	−.017		.006	−2.66	.008	−.029	−.004	***
Constant	24.842		.692	35.87	.000	23.484	26.199	***
Mean dependent var			24.354	SD dependent var		6.128		
Number of obs			1908	Model Chi-square		12739.839		
Prob > Chi-square			0.000	Akaike crit. (AIC)		12281.470		

*** *p*<.01, ** *p*<.05, * *p*<.1.

Abbreviations: ALSFRS-R: Amyotrophic Lateral Sclerosis Functional Rating Scale-Revised; Coef.: coefficient; St.Error: standard error; Sig: significance.

### Change analysis

Change was investigated for those who had completed their first three questionnaires (‘trilogy’ group) over a period of 18.3 months with an average baseline duration since diagnosis of 23.3 months, together with a subset of these followed-up over 13.6 months with an average baseline duration since diagnosis of 1.4 months (‘inception’ group) (Table 7). Baseline levels of ALSFRS-R for both groups were significantly different between the raw score totals and interval measures and likewise the magnitude of change. For the inception group, the average monthly reduction in ALSFRS-R was 0.41 on the interval level metric, and 0.60 on the ordinal raw score. This would equate to a traverse of about 20 points on the interval measure over 48 months (approaching 30 points on the ordinal). The rate of change was higher in the inception group than in the full trilogy group.

**Table 7.** Changes in ALSFRS-R (ordinal and interval) for those completing baseline and first two follow-ups.

	Interval	Ordinal	Time in study (months)	Interval average monthly change	Ordinal average monthly change	Duration since diagnosis (months)	*N*
Trilogy group							
Baseline	27.5	36.4	0			23.3	231
First Follow-up	25.5	33.3	11.1	0.18	0.28	35.7	230
Second Follow-up	23.5	30.5	18.3	0.28	0.40	42.6	230
Baseline to second	−4.0	−5.9	18.3	0.22	0.34		
Inception group							
Baseline	28.8	38.3	0			1.4	108
First Follow-up	25.9	34.3	7.5	0.39	0.53	8.9	107
Second Follow-up	23.2	30.2	13.6	0.44	0.67	14.8	107
Baseline to second	−5.6	−8.1	13.6	0.41	0.60		

## Discussion

Analyzing data from the ALSFRS-R in a large sample of those with ALS initially failed to support the total score from both classical (confirmatory factor analytic) and modern (Rasch analysis) psychometric perspectives. However, applying a bi-factor solution within the Rasch measurement framework generated an interval level total measure, though 18% of the unique variance attributed to the subscales had to be discarded. It was also possible to generate interval level measurement for the Bulbar, Fine- and Gross-Motor subscales, as well as the Limb subscale, which comprises both Fine- and Gross-Motor. The Respiratory subscale cannot generate interval level measurement, but its ordinal score is valid.

These results support previous studies which reported that the ALSFRS-R is multidimensional and that the raw score total should not be used as an endpoint in studies (5, 7). Indeed, the raw score total cannot be recommended for decision making in a clinical setting, at either group or individual level, because inherent multidimensionality renders it invalid even at an ordinal level.

Evidence from the current study shows it is valid to use a total measure based on the bi-factor solution, using the transformed metric via the transformation table provided. The two-testlet solution to produce the total score may reflect that fine/gross motor tasks are under conscious control while respiratory and bulbar functions are involuntary.

In addition, several subscales can be used, specifically Bulbar, Fine- and Gross-Motor (the two Motor can be combined into Limb), either at the ordinal level with appropriate statistics, or at the interval level with parametric statistics following transformation as above.

Currently, the risk of bias from using a less-than-interval and multidimensional total ALSFRS-R score is unknown. However, it has been shown that misusing ordinal scales can produce biased outcomes (24, 25). The current study demonstrates that for the ALSFRS-R, the (inappropriately) calculated raw score change will underestimate change at the margins of the scale, and overestimate change for the central part of the scale. Consequently, where the change largely occurs within the interquartile range of the scale (i.e. ALSFRS-R raw score range 36–12), then it will overestimate the true (metric) change. This is particularly pertinent as, in the inception cohort, the entry level on the ALSFRS-R at diagnosis was 38.3 on the ordinal. This suggests that any deterioration of functioning would occur over the center of the scale, and thus overestimate the decline in function if based on the ordinal raw score.

Figure 4 showed that relatively few raw score points are lost descending from the score of 48 compared to the metric, so leaving the raw score total much higher than the metric at an equivalent level of functioning. It follows that reporting change scores for total raw ALSFRS-R scores is subject to variability depending on the starting point (26). This illustrates that a 1-point change on the ALSFRS-R raw score corresponds to different quantities of change in functioning depending on the starting point and the subsequent range of change; as a consequence mathematical operations on the raw scores such as the calculation of means, change scores or effect sizes are invalid. This problem can be overcome by using the interval level transformations provided in this study, which permit parametric statistics such as means or change scores. To illustrate the impact of the incorrect use of means of ordinal measures, the invalid mean change on the ordinal from baseline to the second follow up was 8.1 compared to 5.6 on the interval level metric.

Acknowledging the limitations of using ordinal measures, to provide some context against existing publications, the monthly average decline (0.60) in the raw score of the inception group is the same as that reported during the first year after diagnosis in a recent Italian population study (27). It is lower than that reported for trial populations, as these seek to exclude slow progressors whose inclusion would increase the duration or sample size of the study (28, 29).

At least two other measures of functioning with interval scale estimates have appeared recently, one an established generic scale (World Health Organization Disability Assessment Schedule 2.0: WHODAS 2.0) (30) with published Rasch transformation (31), and the other a disease-specific scale (Rasch-Built Overall Amyotrophic Lateral Sclerosis Disability Scale: ROADS) (32). Several issues need to be addressed with respect to these scales. Are they measuring the same construct as each other and the ALFRS-R, can their scores be compared to antecedent ALSFRS-R scores for historical comparisons, as well as comparisons of current study outcomes? Both aspects need detailed investigations, but if they are found to be measuring the same construct, Rasch-based strategies can be used to provide a cross-walk between scale estimates (33).

This study has several strengths such as large sample size, use of both a calibration sample to remove time dependency in the estimates derived from the Rasch model, and of training and validation samples to facilitate cross-validation. Both the long period of data collection and the wide range of participating sites should contribute to the generalizability of the findings. Finally, the application of interval scale estimates allows for valid calculations of SEM and SDD, and the use of multi-level regression analysis.

The limitations include the relatively low proportion of those with respiratory onset, even in a large national cohort. The transformation tables should only be used with complete data. However, previous work has indicated that imputation has little effect on fit to the Rasch model, so imputation permits use of the transformation table if item responses are missing (34). ALSFRS-R items such as ventilation partly reflect treatment availability, so healthcare setting might influence use of the scale. Finally, duration used is time since diagnosis.

Future work should include studies of the Minimum Clinically Important Difference (MCID) and comparison of effect sizes between levels of perceived change. In addition, the magnitude of change experienced by patients could be examined with respect to disease progression (35).

The clinical implications are that the use of a raw ALSFRS-R total score in clinical trials, without transformation to interval scaling, may lead to an unknown level of bias (24, 25). In routine clinical monitoring, interpretation of raw score total changes may give the wrong impression of a slow decline at the margins of the scale, and a faster decline across the central part of the scale.

Despite several publications demonstrating the substantial limitations to the measurement properties of the ALSFRS-R, it remains a widely used measure of functional status in ALS/MND. The current study has shown that a total ALSFRS-R ordinal raw score could lead to inferential bias in clinical trial results due to its non-linear nature. Following transformation to interval level metric data, a difference of 5 points is required before a statistically significant detectable difference can be observed. Use of the linear transformation should be mandatory in trials.

## Supplementary Material

Supplemental Material

## Data access

Data supporting this study are not openly available due to reasons of sensitivity and are available from the corresponding author upon reasonable request. Data are located in controlled access data storage at Walton Center NHS Trust. Please contact wcft.tonic@nhs.net.
