# Supplementary material for: Improving the measurement properties of the Amyotrophic Lateral Sclerosis Functional Rating Scale-Revised (ALSFRS-R): deriving a valid measurement total for the calculation of change
Source: Amyotroph Lateral Scler Frontotemporal Degener. 2024 Mar 1;25(3-4):400–9. doi: 10.1080/21678421.2024.2322539 (PMC11262430; doi:10.1080/21678421.2024.2322539)
Supplement: Supplemental Material [file IAFD_A_2322539_SM6790.pdf]

Supplementary File

This supplementary material is from:

Improving the measurement properties of the Amyotrophic Lateral Sclerosis Functional Rating Scale-Revised (ALSFRS-R): deriving a valid measurement total for the calculation of change

Amyotrophic Lateral Sclerosis and Frontotemporal Degeneration Journal 2024. doi: 10.1080/21678421.2024.2322539

Contents

1. Amyotrophic Lateral Sclerosis Functional Rating Scale Revised (ALSFRS-R) Versions. 1
1a. Differences between TONiC patient reported ALSFRS-R and clinician administered Cedarbaum, ENCALS and NEALS versions of ALSFRS-R 1
1b. References 3
2. Rasch Analysis 3
2a. Calibration, Training and Validation Samples for Rasch Analysis 3
2b. Methods of Rasch Analysis 3
2c. Detailed Results of Fit to the Rasch Model 5
Supplementary File: Table 1. Strategies seeking fit of the data to the Rasch model. 6
3. Additional Analyses 7
3a. Confirmatory Factor Analysis (CFA) 7
3b. Reliability 7
3c. References 7

1. Amyotrophic Lateral Sclerosis Functional Rating Scale Revised (ALSFRS-R) Versions

1a. Differences between TONiC patient reported ALSFRS-R and clinician administered Cedarbaum, ENCALS and NEALS versions of ALSFRS-R

The original scale was published by Cedarbaum (1). The European Network to Cure ALS (ENCALS) has published a version (2) as has Northeast Amyotrophic Lateral Sclerosis Consortium (NEALS) (3).

The TONiC patient reported ALSFRS-R was based on that validated by Montes (4). All items match the clinician administered versions, usually without change in wording. Most modifications were minor edits to use lay language.

There were several changes arising from the cognitive debrief. Some addressed the need from patients for clarity of wording. For example, item 11: Orthopnoea (2) - '*needs more than 2 pillows':* patients criticised this if they were using specialist profiling beds to achieve the effect on posture of using more than 2 pillows, so wording was altered to two pillows or an equivalent. Item 2: Salivation - patients thought it hard to distinguish between '*nighttime*', '*minimal*' and '*some*' drooling and found the items clearer if *minimal* and *some* were variations of daytime drooling (*minimal* is so qualified in the ENCALS 2015 version). Clinical phrases such as item 8: (3) Walking - '*Early ambulation difficulties'* were better understood when altered to "Walking has changed but do not require any assistance or devices", which preserved the hierarchy with the item above (normal), and below (walks with assistance). The item requiring complete rephrasing compared to the clinician administered version was item 8: Walking (1) - '*Non-ambulatory functional movement'*, changed to "Can move legs and stand up but unable to walk". Both the original Cedarbaum and ENCALS versions qualify '*during the night'* for item 12: Respiratory insufficiency (2) - '*continuous use of BiPAP*', and we adopted this.

There were two items disparaged by patients. All three clinician administered versions use the same wording for item 10: Dyspnoea (0) - '*Significant difficulty: considering using mechanical respiratory support*'. Patients with significant difficulty who had declined respiratory support wanted the condition of considering mechanical support removed because they could affirm the first clause about difficulty but not the second about considering ventilatory support. For item 11: Orthopnoea (0) - '*unable to sleep'*, patients commented they could not state they were unable to sleep because this must be untrue if they had slept at all. We noted the ENCALS alteration of adding the qualifier '*without mechanical assistance*' and altered this to "Require the use of respiratory (breathing) support (NIV) to sleep" which was clear and correctly answered by all subjects in the pilot study.

1b. References

1. Cedarbaum JM, Stambler N, Malta E, Fuller C, Hilt D, Thurmond B, et al. The ALSFRS-R: a revised ALS functional rating scale that incorporates assessments of respiratory function. BDNF ALS Study Group (Phase III). Journal of the Neurological Sciences. 1999;169(1-2):13-21.
2. ENCALS. ALS Functional Rating Scale Revised (ALS-FRS-R) 2015 [Available from: https://www.encals.eu/wp-content/uploads/2016/09/ALS-Functional-Rating-Scale-Revised-fill-in-form.pdf.
3. NEALS. ALSFRS-R (NEALS): ALS C.A.R.E. Program, Center for Outcomes Research, University of Massachusetts Medical School; [Available from: https://www.outcomes-umassmed.org/ALS/alsscale.aspx.
4. Montes J, Levy G, Albert S, Kaufmann P, Buchsbaum R, Gordon PH, et al. Development and evaluation of a self-administered version of the ALSFRS-R. Neurology. 2006;67(7):1294-6.

2. Rasch Analysis

2a. Calibration, Training and Validation Samples for Rasch Analysis

For Rasch analysis, population representativeness is not a requirement, but rather a wide range of person 'ability' (in this case levels of functional status) is needed (1). A calibration sample of 1000 participants was created consisting of multiple time points where individuals were sampled without replacement, in such a way that an individual is only included once in the calibration sample, to avoid dependency inherent in repeated measures (2). The calibration sample was further randomized into 'training' and 'validation' samples of 500 participants for use in both the CFA and Rasch analysis. The sample size of 500 was geared to maintaining a Type I error rate of 5% for the Rasch fit statistics (3, 4).

2b. Methods of Rasch Analysis

Data from each (sub)scale was tested against the requirements of the Rasch Measurement model (5). Briefly, these requirements include: i) unidimensionality; ii) monotonicity; iii) homogeneity; iv) local independence and v) group invariance (6, 7). Whichever set of items are to be added together to provide a score, they should satisfy all these requirements. They should: i) measure one thing (domain/construct/trait; ii) the probability of a positive response to an item (or in the case of polytomous items, the transition from one response category to the next) should increase with underlying

ability, as should the total score (8); iii) the same hierarchical ordering of items should hold for each level (or grouping) of the score (9); iv) items should be conditionally (on the score) independent of one another (10); and v) the response to items across different groups such as age or gender should, conditioned on the total score, be the same – referred to as (the absence of) Differential Item Functioning (DIF) (7). Whichever set of items are to be added together to provide a score, they should satisfy all these requirements.

Each requirement is tested. A t-test is used to determine if two separate groups of items deliver significantly different estimates, following the procedure given by Smith (11)**.** The hierarchical ordering of items across the scale is determined through a Chi-Square test of fit based on grouped scores. Monotonicity is evaluated through inspection of the item-category ordering. Conditional item dependence is determined though the correlation of residuals, where pair-wise correlations should not exceed 0.2 above the average residual (12)**.** Where pairs of items were identified as locally dependent, they were merged into super items (i.e. identified *post-hoc* from the residual correlations). For item sets with subscale structures, the items can be grouped as subscale testlets, simply adding them together to make one larger item to absorb the local dependency (i.e. an *a priori* definition) (13). In the RUMM2030 software, this gives a bi-factor equivalent solution retaining a specified proportion of the variance. This "Explained Common Variance (ECV)" is reported, whereby a value less than 0.7 is indicative of requiring a multidimensional model, a value above 0.9 a unidimensional model, and the grey area in between, undetermined, requiring further evidence (14)**.** Consequently, value of the ECV at 0.9 and above is considered acceptable in the current analysis. If two parallel forms are created from either a subscale structure, if present, or from the pattern of local dependency in the item set, this requires a latent correlation ≥ 0.9. This is consistent with the reliability required for individual use (15). Consequently, valid parallel forms would require both their latent correlation to be ≥0.9 and the ECV to be ≥0.9.

The scales were also tested for invariance (differential item functioning -DIF); DIF occurs when subjects on the same level of the latent trait, such as disability, answer the same item differently depending on their group memberships (e.g. age, gender) (16). DIF was examined for a series of contextual factors including age, gender, time-

point (repeated measurement), duration of ALS (grouped into quartiles) and onset type (limb, bulbar or respiratory). Should DIF be identified it is tested by a comparison of person estimates from split and unsplit solutions to see if it is ‘substantive’ (17). Where the difference is significant (a paired t-test), the result is reported as an effect size where a value higher than 0.1 is considered to represent substantive DIF (18). If this is present, then the scale works in different ways for the contextual factor under consideration, and results are reported separately. Finally, reliability is reported as both a Person Separation Index (PSI), and as Cronbach’s alpha. If data are normally distributed they are equivalent, but otherwise PSI tends to be lower the more data are skewed. Values are treated the same, and so values below 0.7 would be described as low, as they do not support group use.

A hierarchical approach to seeking fit of the data to the model for existing scales is adopted, with level 1 as the priority (Supplementary File: Table 1). All aspects listed above must be met. Should a level 5 solution be unavailable, item deletion will be considered (level 6). If this fails then level 7 will be utilised to test if the scale satisfies ordinal scaling; if not level 8 remains, indicating failure.

2c. Detailed Results of Fit to the Rasch Model

The Bulbar, Fine-Motor and Gross-Motor subscales each achieved acceptable fit to the model, with occasional DIF which was not replicated across samples. The Gross-Motor scale displayed local item dependency between two variables (‘walking’ and ‘climbing stairs’), which were made into a single super item. Note that 17% of the variance had to be discarded to make a unidimensional latent estimate of the Gross-Motor domain (ECV 0.83). While the Limb domain (comprising both Fine- and Gross-Motor function) had acceptable fit, it displayed gender DIF in both ‘training’ and ‘validation’ samples. However, the effect size of the comparison of estimates from a split and unsplit solution was just 0.02, and thus no further action was taken. The Respiratory subscale did not show adequate fit in either sample.

A total score achieved satisfactory fit to the model under a bi-factor solution, based upon two testlets, one containing all the items from the Bulbar and Respiratory subscales, and the other containing the remaining Limb (Fine-Motor and Gross-Motor)

domain items. In the validation sample, 15% of the variance had to be discarded to achieve this solution.

Supplementary File: Table 1. Strategies seeking fit of the data to the Rasch model.

Level	Nature	Adjustments	Reporting		
			Chi-Square	ECV ≥0.9	Latent Correlation ≥0.9
1	Item-based	None	Interaction	No	No
2	Item-Based	Clusters for Local Item Dependency	Interaction	Yes	No
3	Domain-based	On existing sub-scales >2	Interaction	Yes	No
4	Parallel Form	On existing sub-scales ≤2 or<br>2 local dependency patterns or conceptual groups	Conditional	Yes	Yes
5	Parallel Form	On alternative items	Conditional	Yes	Yes
6	Item Deletion	On reduced items, and repeating levels 1-5	Interaction	No	No
7	Mokken Scaling	On items if Unidimensional. Loevinger's coefficient H ≥0.4-moderate	No	No	No
8	Fail	No valid ordinal scale	No	No	No

DIF was also present for onset type but the effect size of the difference between estimates was just 0.019 and so no further action was taken. When the full calibration sample was applied to the total score, 18% of the variance had to be discarded to achieve a unidimensional latent estimate, given model fit. Parameters from any valid solution within the calibration sample were then imported into the main data set to obtain the most accurate estimates for the ALSFRS-R.

3. Additional Analyses

3a. Confirmatory Factor Analysis (CFA)

Confirmation of fit required a non-significant Chi-Square (χ^2) (particularly with this sample size), with approximate fit statistics in support (19). The latter include the Root Mean Square Error Approximation (RMSEA) (required value <0.6), Confirmatory Fit Index (CFI) and Tucker Lewis Index (TLI) (required values ≥0.95). Given the nature of health assessments, where often items represent nuances of the construct, giving rise to the failure of the local item independence assumption, item errors were allowed to be correlated within their respective domains.

3b. Reliability

Reliability was determined from two measures, Cronbach's alpha and the Person Separation Index (PSI). Cronbach's alpha is a function of the number of items in a test, the average covariance between pairs of items, and the variance of the total score. It is calculated on the ordinal, whereas the PSI is the same, but calculated on the interval. The PSI is more sensitive to skewed distributions.

3c. References

1. Andrich D: Rasch models for measurement. California: Sage Publications 1988.
2. Mallinson T. Rasch Analysis of Repeated Measures. Rasch Measurement Transactions. 2011;25(1):1317.
3. Hagell P, Westergren A. Sample Size and Statistical Conclusions from Tests of Fit to the Rasch Model According to the Rasch Unidimensional Measurement Model (Rumm) Program in Health Outcome Measurement. J Appl Meas. 2016;17(4):416-31.
4. Müller M. Item fit statistics for Rasch analysis: can we trust them? Journal of Statistical Distributions and Applications. 2020;7(1):5.
5. Rasch G. Probabilistic Models for Some Intelligence and Attainment Tests. Chicago: The University of Chicago Press; 1980.
6. Gustafsson J. Testing and obtaining fit of data to the Rasch model. British Journal of Mathematical & Statistical Psychology. 1980;33(2):205-33.
7. Teresi JA, Kleinman M, Ocepek-Welikson K. Modern psychometric methods for detection of differential item functioning: application to cognitive assessment measures. Stat Med. 2000;19(11-12):1651-83.
8. Kang HA, Su YH, Chang HH. A note on monotonicity of item response functions for ordered polytomous item response theory models. The British journal of mathematical and statistical psychology. 2018;71(3):523-35.
9. Rost J. An unconditional likelihood ratio for testing item homogeneity in the Rasch model. Education Research and Perspectives. 1982;9(June):7-17.
10. Wilson M. Detecting and Interpreting Local Item Dependence Using a Family of Rasch Models. Applied psychological measurement. 1988;12(4):353-64.

11. Smith E. Detecting and evaluating the impact of multidimensionality using item fit statistics and principal component analysis of residuals. J Appl Meas. 2002;3:205-31.
12. Christensen KB, Makransky G, Horton M. Critical values for Yen's Q3: Identification of local dependence in the Rasch model using residual correlations. Applied psychological measurement. 2017;41(3):178-94.
13. Wainer H, Kiely G. Item clusters and computer adaptive testing: A case for testlets. J Educ Meas. 1987;24(3):185-202.
14. Quinn H. Bifactor Models, Explained Common Variance (ECV), and the Usefulness of Scores from Unidimensional Item Response Theory Analyses [Masters Thesis]. North Carolina: University of North Carolina at Chapel Hill; 2014.
15. Bland JM, Altman DG. Statistics notes: Cronbach's alpha. British Medical Journal. 1997;314:572.
16. Holland PW, Wainer H. Differential item functioning: Psychology Press; 1993.
17. Hagquist C, Andrich D. Recent advances in analysis of differential item functioning in health research using the Rasch model. Health Qual Life Outcomes. 2017;15(1):181.
18. Rouquette A, Hardouin JB, Vanhaesebrouck A, Sébille V, Coste J. Differential Item Functioning (DIF) in composite health measurement scale: Recommendations for characterizing DIF with meaningful consequences within the Rasch model framework. PLoS One. 2019;14(4):e0215073.
19. Kline R. Principles and Practice of Structural Equation Modeling. Third Edition ed. New York, London: Guilford Press; 2011.
